# Hierarchical decision-making produces persistent differences in learning performance

**DOI:** 10.1038/s41598-018-34128-w

**Published:** 2018-10-25

**Authors:** Thorbjørn Knudsen, Davide Marchiori, Massimo Warglien

**Affiliations:** 1 Strategic Organization Design unit and Danish Institute for Advanced Study, University of Southern Denmark, Odense, Denmark; 2 Department of Management, Ca’ Foscari University of Venice, Venice, Italy

## Abstract

Human organizations are commonly characterized by a hierarchical chain of command that facilitates division of labor and integration of effort. Higher-level employees set the strategic frame that constrains lower-level employees who carry out the detailed operations serving to implement the strategy. Typically, strategy and operational decisions are carried out by different individuals that act over different timescales and rely on different kinds of information. We hypothesize that when such decision processes are hierarchically distributed among different individuals, they produce highly heterogeneous and strongly path-dependent joint learning dynamics. To investigate this, we design laboratory experiments of human dyads facing repeated joint tasks, in which one individual is assigned the role of carrying out strategy decisions and the other operational ones. The experimental behavior generates a puzzling bimodal performance distribution–some pairs learn, some fail to learn after a few periods. We also develop a computational model that mirrors the experimental settings and predicts the heterogeneity of performance by human dyads. Comparison of experimental and simulation data suggests that self-reinforcing dynamics arising from initial choices are sufficient to explain the performance heterogeneity observed experimentally.

## Introduction

Human organizations are commonly characterized by a hierarchical chain of command that facilitates division of labor. Higher hierarchical levels set decision premises that guide, constrain, and focus the operational tasks carried out by lower-level employees. This scheme, a cornerstone in modern organization theory^1^, is adopted by most human organizations, independent of ownership, size, and purpose. For this reason, decision-processes in most human organizations occur at multiple levels that operate on information with different degrees of abstraction, and at different time scales.

A fundamental motivation for hierarchically distributed decision processes is that they overcome individual cognitive limitations by integrating contributions from multiple actors to facilitate effective organizational responses (e.g.^1–5^). While hierarchical decision processes may facilitate effective adaptive responses, they also promote feedback ambiguity which complicates experiential learning^6–8^. Whenever decisions produce unexpected outcomes, it is unclear whether such outcomes are due to environmental noise, to mistakes made by lower level actors, or to wrong decision premises set by actors at the higher level. As the dynamics of nuclear accidents dramatically demonstrate, wrong decision premises can make it extremely hard to interpret and identify the causes of error. For example^9^, argues that major accidents as the Chernobyl disaster were the result of the interaction of active errors, made by front-line operators, and latent errors, which are hidden in decision premises set by higher level supervisory and design roles. Thus, hierarchical decision-processes can relieve the effects of individual cognitive limitations, but they can also increase the ambiguity of feedback and complicate learning from experience.

Hierarchical decision processes are not unique to social organizations. Recent research has shown that the nature of the neurophysiological processes at the basis of individual perception, cognition, and decision-making are hierarchical and occur at multiple time-scales^10–17^. Authors of^13^ focus on individual hierarchical decision processes that can be categorized into (high-level) strategy and (low-level) operational decisions, operating at different timescales. They show that, because of the difficulty in disambiguating the source of decision errors, critical two-way interactions occur between choices at the operational level and the selection of strategies at the higher level.

In this paper, we explore a problem structurally analogous to that considered in^13^, with the difference that it is contextualized in the domain of social organizations. In our study, the hierarchical decision processes do not occur within the same individual, but are distributed across different individuals. In a behavioral study and a hybrid model of multi-level learning, we examine the experimental behavior of hierarchical dyads, in which the high-level agent (henceforth H-agent) carries out strategy decisions, and the low-level agent (henceforth L-agent) ongoing operational decisions. Our computational model reproduces the organizational architecture and the learning processes occurring in the experiment with human participants. Both human and simulated dyads of agents have to learn how to evaluate a sequence of randomly generated multidimensional numerical vectors (*inputs*) that can be interpreted as input information coming from the external environment.

Our design captures the three basic properties of hierarchical interaction. First, the two agents operate over different time scales, as one strategy-decision by the H-agent is followed by multiple operational decisions by the L-agent. Second, the H-agent is responsible for selecting a proper subset of the input components (strategy decisions) that best enables the L-agent to evaluate each input (operational decisions). Third, the H-agent receives feedback about the L-agent’s performance, whereas the L-agent evaluates each input based on the subset of input components that are accessible to her within the frame set by the H-agent. Three main assumptions of our design make the task of the L- and H-agents non-trivial: (i) input components vary with respect to the informative value that they provide to the L-agent; (ii) the L-agent evaluates each input only based on the knowledge of a proper subset of input components that the H-agent makes available to her; (iii) the informative value of the input components is unknown to both agents: The H-agent must learn to disclose the most informative input components solely based on the feedback from the L-agent’s performance.

Our hybrid hierarchical model of dyad learning parsimoniously blends two different learning approaches (e.g.^18^; see SI for a comparison with models that include a larger number of free parameters and/or assume more sophisticated learning processes), since the H- and L-agents carry out different tasks and receive different feedback. Rather than providing the best model for describing behavior in hierarchical settings, our model provides a plausible explanation for the experimentally observed phenomena (see SI sections on the model’s goodness of fit and on model comparison). The L-agent’s model reflects a supervised learning task that can be equated to a classical function learning problem, i.e., that of assessing the value of a sequence of stimuli (in our case, multidimensional numerical inputs). Thus, our model for the L-agent is based on the “delta rule,” a core rule of connectionist models of human learning (ref.^19^, on neural nets; see also^20^ and^21^ for applications to multi-agent learning) and attention allocation^22^. In contrast, the H-agent learns to choose, in an unsupervised way, among a finite set of strategies (i.e., the ways of selecting a proper subset of the components of an input), and can only rely on feedback about the L-agent’s performance conditional on the strategy previously chosen. Reinforcement learning, the model we use for the H-agent, has been shown to be very effective in describing learning processes when feedback is limited to rewards obtained from the selected action^18,23,24^.

## Results

The experiment is designed to reproduce a dyadic hierarchical interaction with the features described earlier. The goal of each dyad of participants was to accurately estimate the value of the input, which is the weighted sum of the triplets of numbers of the input, without knowing the value of one input component and without receiving any information about the vector of weights, which had to be learned. Participants were only told that each weight is a number between 0 and 1, that weights do not necessarily sum up to 1, and that they are kept fixed all throughout the experiment (cf. experimental instructions in SI). Since the vector of weights is set to {0.9, 0.5, 0.1} (up to a random permutation for each dyad), input components have distinct contributions to the input’s target value.

**Figure 1:**
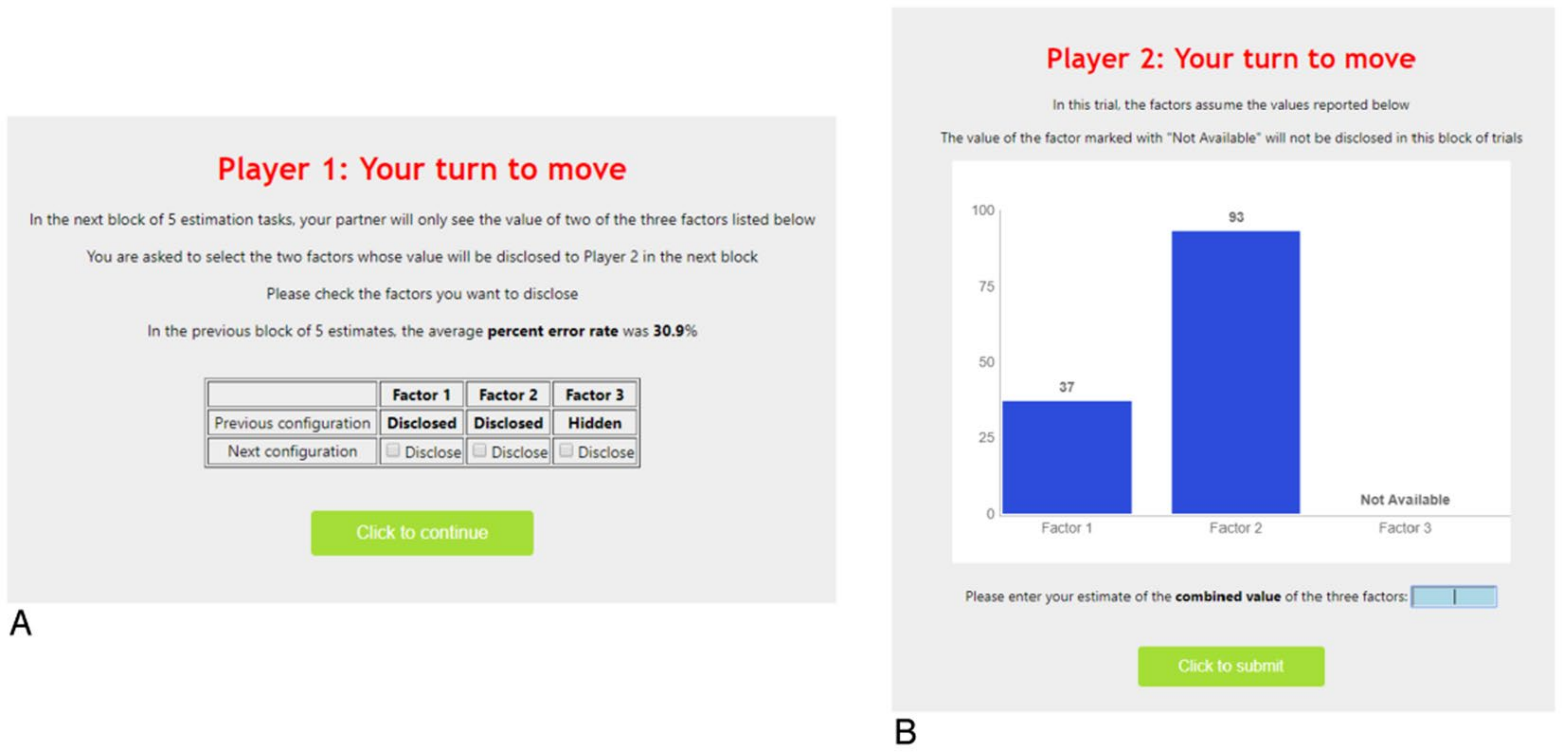
Screenshots from the experiment. These screenshots from the experiment give an example of the task faced by the H-agents (Panel (A)) and L-agents (Panel (B)). These screenshots were included in the instructions that were handed and read aloud to the participants before the starting of each experimental session (see SI).

**Figure 2:**
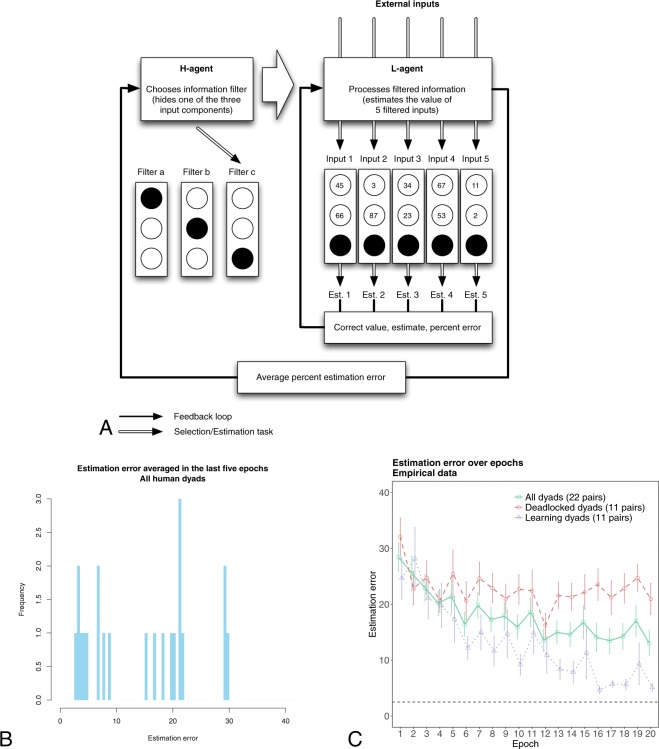
Structure of the interaction between the H- and L-agent and main empirical results. Panel (A) illustrates the structure of interaction between the H- and L-agent. Panel (B) shows the distribution of estimation error in the last five epochs, whereas panel (C) shows the trajectory of the estimation error over epochs. In panel (C), error bars indicate the 95% normal confidence intervals. The lowest attainable average estimation error (panel (C), horizontal dashed line) is attained when the H-agent selects the optimal information filter (i.e., the one that hides the component with the lowest weight) and the L-agent has converged to the correct weights. Thus, under this condition, the correct input value and the estimated value diverge by 0.1 *E|X - c|, where X ~ U[0, 100] is the value of the hidden input component, and c the guess of its value by the L-agent: Given this observation, the optimal guess for the L-agent is 50 (the median, and mean, of X), which implies a value of 2.5 for the lowest attainable average estimation error.

The H-agent moves first and applies a filter to the incoming environmental information (inputs). Specifically, she selects which two of the three input components will be disclosed to the L-agent: This is the “decision premise” for the L-agent’s operational tasks. In this setting, the H-agent can choose among three possible alternatives (or information filters)–the number of possible ways for disclosing two out of the three input components. For concreteness, one can interpret such decision premise as providing a focus of attention that benefits the L-agent because of her limited processing capabilities. Since the different input components differently contribute to the input target value, the filter that provides the best premise for the L-agent’s estimation tasks (the “optimal filter”) is the one that hides the input component with the smallest weight–i.e., 0.1. Subsequently, the L-agent estimates in sequence five different filtered inputs, therefore by knowing the value of only two input components (see the screenshots from the experiment reported in Fig. 1 and the graphical summary of the experimental design in Fig. 2A). We refer to an *epoch* as a sequence encompassing one selection of the information filter (carried out by the H-agent), followed by a sequence of five estimation tasks (carried out by the L-agent).

Our choice of considering tri-dimensional input vectors and filters that hide one of the input components might appear artificial, but it is strongly motivated by results from a second, similar experimental study (described in SI). In this second study, the constraint on information disclosure was relaxed: The H-agent could, at a cost, decide to disclose to the L-agent all three input components at any given epoch. This choice of full disclosure was selected in about 22% of the epochs. It turns out that the full disclosure of input components confuses the L-agents rather than facilitating their learning process: When presented with all three input components, L-agents’ estimation error is significantly higher than when presented with only two. This observation is consistent with the psychological literature on information overload and its effects on attention^25–27^.

As characteristic of hierarchies, in our design, feedback is different for the two roles. For both agents, we use estimation error–i.e., the absolute distance between the actual and the estimated value of an input–as a fundamental measure of performance, and thus feedback (see Methods). This measure differs in coarseness for the two agents. Starting from the second epoch, the H-agent receives feedback corresponding to the L-agent’s average percent estimation error in the previous epoch. In contrast, the L-agent receives more detailed feedback. After each estimation, the L-agent observes the actual value of the input, and thus receives feedback about the sign and magnitude of the estimation error.

The details about participants, input target values, timing of the interaction, and feedback are reported in the Methods.

In our analysis, we use the estimation error as the measure for dyadic performance. This error measure reflects the joint learning processes that encompass strategy and operational decisions, rather than the accuracy of the L-agent alone. This is because the H-agent’s decisions affect the maximal level of accuracy that the L-agent can possibly attain.

The estimation error over epochs averaged over all dyads displays a classical learning curve (Fig. 2C). A two-way ANOVA analysis with a repeated measures factor shows a significant epoch effect (F[19, 380] = 4.11, p < 0.001). However, dyads’ learning curves are rather heterogeneous, suggesting a disaggregated analysis of the learning trajectories. The distribution of the estimation error averaged over the last five epochs, illustrated in Fig. 2B, displays a puzzling bimodal shape: A Hartigan’s dip test rejects the null of unimodality (D = 0.1062, p = 0.03), suggesting that dyads’ performance in the long term can be meaningfully clustered into two different groups. Indeed, for about half of the dyads (ten) the estimation error in the last five epochs is smaller than ten, and the H-agents in these dyads always selected the optimal filter in the last five epochs. As learning processes usually generate unimodal distributions of outcomes, a natural question is whether the unusual bimodal outcome may be a result of the hierarchical nature of decision making.

In light of these observations, we split the 22 dyads into two groups according to the median value, 16.1, of the estimation error averaged over the last five epochs. We refer to these two groups as “learning dyads” (the eleven dyads for which the estimation error in the last five epochs is smaller than its median), and “deadlocked dyads” (the remaining eleven dyads). The decision to operate a median split has at least three motivations. First, it ensures that the ensuing two subgroups include a balanced number of observations that allows for meaningful statistical testing. Second, it provides a simple rule for clustering dyads that allows for the comparison of results across experimental and simulation studies (cf. Study 2 described in SI). Third, the median split is coherent with the distribution of the estimation error in the last five epochs, as it groups together the ten dyads that converged to the selection of the optimal filter together with one dyad in which the H-agent selected the optimal filter in four out of the last five epochs.

The two groups of dyads exhibit significantly different patterns of learning: In an ANOVA, both the group factor and the epoch-group interaction are significant (respectively, F[1, 20] = 40.97, p < 0.001, and F[19, 380] = 2.08, p = 0.005). Whereas a median split is trivially expected to produce significant differences between the two ensuing subgroups, this should not overshadow the result that these subgroups exhibit qualitatively opposite learning patterns. Learning dyads keep improving performance over time and approach optimal behavior at the end of the experiment (for these dyads, the epoch effect is significant, F[10, 19] = 4.68, p < 0.001). In stark contrast, deadlocked dyads lose their adaptive capabilities after a few epochs, and are not able to reduce significantly the error of their estimations over time (for these dyads, the epoch effect is not significant, F[10, 19] = 1.17, p = 0.29). These observations beg a closer examination of the distinct processes that characterize the behavior of H- and L-agents in the deadlocked and learning dyads.

### Disaggregate learning dynamics

The task of the H-agent is to provide the most informative input components to the L-agent. As the H-agent does not know the informational value (weight) of each input component, she must learn it by experimentation, i.e., by observing how the selection of different filters affects the accuracy of the estimates given by the L-agent. A measure that captures such behavior across epochs is the “rate of disclosed information,” defined as the sum of the weights of the disclosed input components, normalized by the sum of the three weights (optimal rate: 0.93; minimal rate: 0.40; random rate: 0.67).

**Figure 3:**
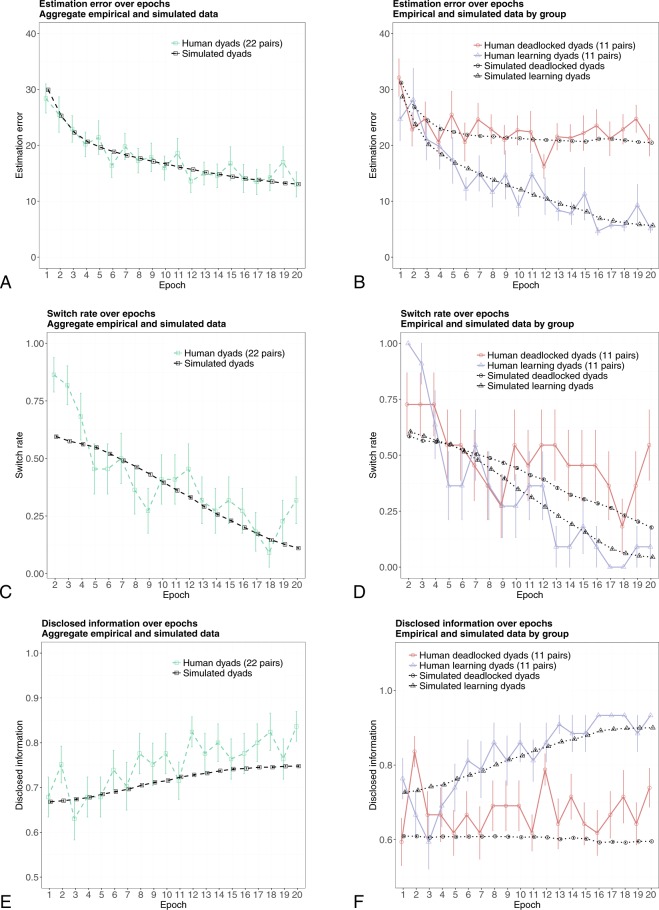
Empirical and simulated results. Panels (A, C and E) show the main empirical and simulated measures of learning performance averaged over all pairs. The aggregate empirical data displayed in panels A and C have been used to fit the HL model’s parameters. Remarkably, under the same parameter values, the HL model is able to predict accurately the learning heterogeneity observed empirically for the estimation error and the switch rate (Panels (B and D)), as well as the rate of disclosed information that was not used to fit the model parameters (**E** and **F**). Error bars indicate the 95% normal confidence intervals.

Figure 3E,F show the rate of disclosed information averaged over epochs, for all 22 dyads and separately for the learning and deadlocked dyads. A two-way ANOVA with a repeated measures factor shows that the group factor, the epoch factor, and the epoch-group interaction, are all significant (respectively, F[1, 20] = 23.79, p < 0.001; F[19, 380] = 2.24, p = 0.002; and F[19, 380] = 2.42, p < 0.001). For H-agents in deadlocked dyads, the rate of disclosed information after epoch 2 is approximately random (0.67) all throughout the experiment. Although the H-agents in the deadlocked dyads provide the L-agents with the most informative input components in more than 20 percent of the times, they subsequently shift to worse information strategies. In contrast, the H-agents in learning dyads approach an optimal rate of disclosed information (0.93), which is attained at epoch 15.

The dyadic learning dynamics is better understood when two additional measures are considered, the H-agent’s sensitivity score, and the H-agent’s switch rate. The H-agent’s sensitivity score measures to what extent the choice behavior by the H-agent at epoch t is affected by fluctuations in the error feedback at epoch t-1 as compared to the previous periods’ estimation error. This score is obtained as the difference between: i) the average estimation error antecedent the selection of a filter different than that selected at epoch t-1 (a *switch*), and ii) the average estimation error antecedent the selection of the same filter selected at epoch t-1 (a *repetition*). Sensitivity scores (which could not be computed for the three deadlocked dyads in which the H-agent always selected the same filter) show that the estimation error before a switch (39.6, SD = 7.19) is significantly higher than that before a repetition (26.1, SD = 24.01) (t[18] = 2.24, p = 0.04). However, the observed aggregate effect is driven by H-agents in learning dyads: For these individuals, the sensitivity score is significantly positive (Mean = 29.1, SD = 8.8; t[10] = 10.95, p < 0.001), and significantly higher than that of H-agents in deadlocked dyads (Mean = −5.4, SD = 34.3; t[17] = 3.23, p < 0.01). In addition, the sensitivity score for H-agents in deadlocked dyads is not significantly different from zero (t[7] = 0.45, p = 0.67).

A second indicator–the switch rate–is defined as the proportion of times in which the H-agent has changed her previous choice throughout the experiment. The switch rate is expected to affect the L-agent’s performance, as less frequent changes in the H-agent’s choice will give her more chances to learn the estimation task. In contrast, frequent switches are likely to confuse the L-agent. Figure 3D suggests different trends for the switch rate in the learning and deadlocked dyads. Although the overall group effect is not significant (F[1, 20] = 2.33, p = 0.14), the switch rate averaged over the last five epochs is significantly different across learning and deadlocked dyads (respectively, 0.05, SD = 0.18, and 0.38, SD = 0.35; t[20] = 2.75, p = 0.01). In contrast, the switch rate averaged over the first five epochs is not different across the two groups (respectively, 0.73, SD = 0.21, and 0.68, SD = 0.34; t[20] = 0.38, p = 0.71). That is, differences between the switch rates of deadlocked and learning dyads emerge during the later stages of the learning process.

Taken together, these indicators suggest that H-agents in learning dyads are more sensitive to payoff variation, and more quickly identify and hold on to the optimal information filter (i.e., that disclosing the input components with weights 0.5 and 0.9). Thus, H-agents in learning dyads appear to be offering their L-agents a better and more stable environment in which to learn. A possible explanation is as follows. If H-agents initially experience unsatisfactory performance, they may start wandering among options as they look for improvement. Such wandering behavior would never produce a stable environment in which L-agents can learn and, in turn, would hinder learning by the H-agent. As a result, both H- and L-agents would be locked into a vicious circle in which they confuse each other, undermining joint learning. This conjecture suggests that the observed differences in learning trajectories reported above can be explained by joint learning dynamics, even without a need to introduce more sophisticated, and perhaps also more realistic behavioral assumptions about H-agents.

Also the differences in sensitivity to feedback and switch rates observed across learning and deadlocked dyads are better understood as the consequence of the outlined joint learning dynamics.

In summary, the data from our experiment show that the hierarchical interaction of decision processes gives rise to learning dynamics that are quite heterogeneous, and leads to performance levels that can be sensibly clustered into two subgroups and that persist in the long run. We propose a computational model of hierarchical interaction that provides sufficient conditions for these phenomena. In particular, the *vicious/virtuous circles* that arise from early interactions between the simulated H- and L-agents accurately replicate those observed experimentally. In addition, the model helps understand better the observed processes of joint learning by disentangling the learning by the two agents.

### A model of hierarchical learning

We develop a computational model of multi-level learning in which artificial dyads of agents face exactly the same task as the human participants do in the experiment. In particular, the weights of input components, the number of epochs and trials per epoch, the feedback received by the two agents, and the way inputs were generated are the same as in the described experimental settings.

**Figure 4:**
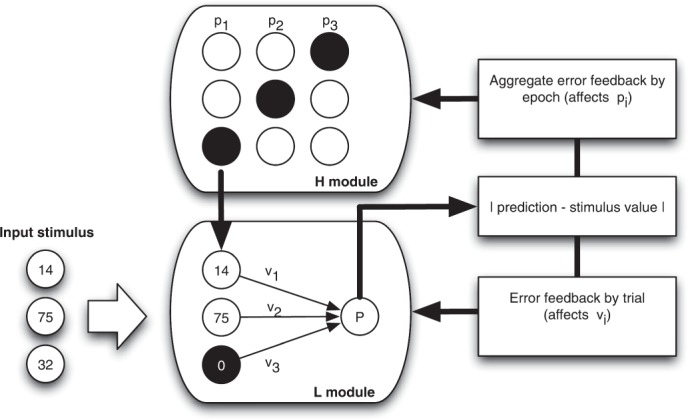
Architecture of the HL learning model. The model consists of two modules: The H-module that carries out the task of selecting among different information filters, and the L-module that carries out the estimation tasks. Whereas the H-module implements the (unsupervised) reinforcement learning algorithm, the L-module is an implementation of the (supervised) “delta-rule” algorithm. The structure of interaction between the H- and L-modules reproduces the one in the experiment with human dyads.

The differences in the feedback received affect the way learning is modeled in each role. As the H-agent receives only payoff-related feedback, we model the H-agent’s learning via an unsupervised, reinforcement learning rule (equation 3 of Methods). Differently from the H-agent, the L-agent receives more detailed feedback and can compare the inputs’ target value with the estimated one. We capture this situation by modeling the L-agent’s learning process as a supervised learning function, via the delta rule (equation 5 of Methods). Thus, our learning model is both hierarchical and hybrid, and we will henceforth refer to it as the “HL-learning” model (Fig. 4).

Figure 3A,C show how HL-learning model fits the experimental data (the parameter estimation procedure is described in Methods). The model captures trends in the experimental aggregate performance of pairs as measured by the empirical estimation error. All but two simulated average values fall within the 95% confidence interval of the corresponding empirical ones (see SI, section 1.3 for more detailed information on the model fit).

**Figure 5:**
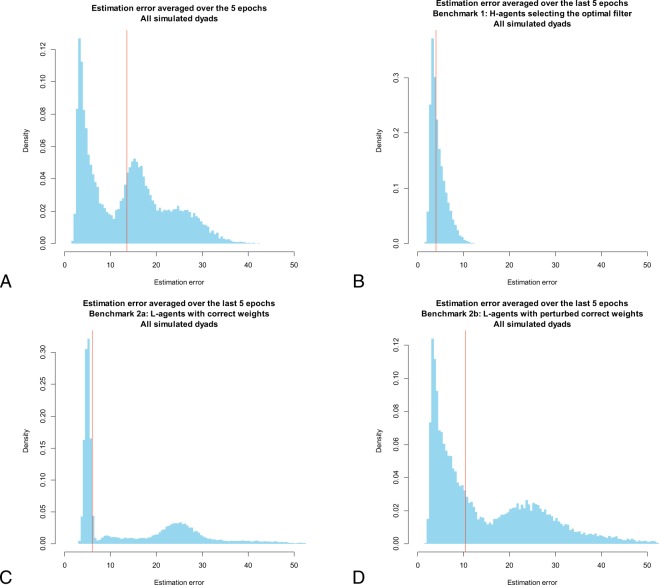
Estimation error averaged over the last five epochs of the simulated experiment. Panel (A) illustrates the distribution of the estimation error averaged over the last five epochs that emerges from the simulation of the baseline model, whereas panel (B) illustrates its distribution when the learning by the H-agents is inhibited and they always select the optimal filter. Panels (C and D) refer to the case in which it is the learning by the L-agents which is inhibited: They are initialized with either the correct weight values (Panel (C)), or with weight values obtained by adding noise to the correct ones (Panel (D); here, noise is a random draw from the U[−0.1; 0.1] distribution). In the simulations reported in Panels (B–D), the learning counterpart uses the best-fit parameters that generated the simulated data reported in Fig. 3. In all panels, the vertical red line represents the median of the distribution.

Remarkably, although the model parameters were solely estimated on the basis of aggregate data obtained from the laboratory experiment, the HL-learning model predicts the heterogeneity in the dyads’ learning curves observed in the lab experiment (Fig. 3B,D,F). The heterogeneity in learning curves obtained from the HL-model is derived from exactly the same procedure used to analyze empirical data from the laboratory experiment: The model parameters are estimated on the aggregate empirical trajectories illustrated in Fig. 3A,C, and the simulated runs under these parameter estimates are split into two groups–one including the artificial dyads with estimation error averaged over the last five epochs above its median, and the other including the remaining dyads. Similar to what we observed for the experimental data illustrated in Fig. 2B, a Hartigan’s dip test rejects the unimodality of the distribution of simulated estimation errors in the last five epochs (D = 0.031, p < 0.001; Fig. 5A). Therefore, as did the laboratory experiment, the HL-model produces two distinct groups of learning curves: One group displays steady learning, whereas for the other, learning deteriorates after a few epochs.

Similar remarks apply when focusing on the learning process that can be observed from simulated H-agents. Again, the model captures the trend of empirical data on disclosed information, as well as the empirically observed trajectories of the H-agents’ switch rates (Fig. 3D,F). The model also predicts the observed divergent trajectories of the learning and deadlocked dyads for all performance measures (Fig. 3B,D,F). See SI, section 3.2, for a more detailed analysis of the predictive performance of the model.

The results on the H- and L-agents’ behavior are especially relevant since our simulated H- and L-agents are identically initialized at the beginning of each simulation–in particular, they shared the same initial behavioral propensities and speed of learning. The divergent trajectories for learning and deadlocked dyads are thus entirely endogenous to the learning process. Early interactions affect the behavior of the H-agent, generating deadlocked dynamics. Similar dynamics suggest a parsimonious explanation of performance heterogeneity observed experimentally, although, of course, other psychological processes such as overconfidence of subjects^8,28^ or their difficulty to take the perspective of the other player^29,30^, may contribute to the empirically observed dynamics.

A better understanding of the vicious circles that trap agents in deadlocked dyads is provided by the analysis of the H- and L-agent’s separate contribution to the simulated dyadic performance. Figure 5 shows the distribution of the estimation error in the last five epochs of the simulated experiment when learning by one of the two agents is inhibited. When the H-agents are constrained to always select the same filter (the optimal one, in the case illustrated in Fig. 5B), the L-agents learn to carry out their estimation task, eventually producing small estimation errors, and the median error (4.0) approaches the lowest attainable one (2.5; cf. the caption of Fig. 2). In this case, the distribution of the estimation error in the last five epochs is clearly unimodal (Hartigan’s dip test, D = 0.0009, p = 1). On the other hand, when the L-agents are initialized with the correct weights and their learning is inhibited, only a subset of the H-agents are eventually able to converge to the choice of the optimal filter, producing a non-unimodal distribution of the estimation error in the last five epochs (Fig. 5C) (Hartigan’s dip test, D = 0.043, p < 0.001), which resembles that of the baseline model with joint learning (Fig. 5A). Indeed, although the L-agents always use correct weights in their computations, their estimations are nonetheless affected by random errors because they are computed based on the value of two of the three input components; such noise in the feedback to the H-agents prevents their learning to always converge to the optimal filter (a well-known property of reinforcement learning in multiple-choice tasks with stochastic, partial feedback). Indeed, perturbing the L-agents’ correct initial weights increases the share of H-agents that do not converge to the optimal filter (Fig. 5D), producing a distribution of the estimation error in the last five epochs closer to that of the baseline model (Fig. 5A).

It is interesting to note that whereas almost all learning dyads in the last five epochs converge to the selection of the optimal filter, the choice behavior of a considerable share of deadlocked dyads does not converge to a unique choice, but select all three filters with some positive probability. According to the HL-model (simulations reported in Fig. 3), 86% of the learning dyads converge to the optimal filter, whereas only 58% of the deadlocked dyads converge to one of the suboptimal choices. The non-converging deadlocked dyads on average select the optimal filter 24% of the time, and 38% of the time each of the other two filters. A similar pattern characterizes experimental data: Ten out of the eleven learning dyads always select the optimal filter in the last five epochs, whereas three of the eleven deadlocked dyads converged to either the second- or third-best filter. In addition, the non-converging deadlocked dyads select the best filter 40% of the time, 38% of the time the second best filter (i.e., the one that hides the input component with weight 0.5), and 22% of the time the worst filter (i.e., the one that hides the input component with weight 0.9). This observation rules out the problem of insufficient exploration (phenomenon largely discussed in psychology; see, for example, ref.^31^) as the main reason for dyadic suboptimal performance, and instead supports our vicious circles hypothesis.

A clear pattern emerges from simulations. After some initial experimentation, H-agents in learning dyads accidentally find the optimal set of input components and are then able to hold on to it for a period of time sufficient for L-agents to significantly reduce their estimation error. These dynamics induce H-agents to stabilize their choice, which, in turn, allows L-agents to further improve their learning of the task, thus reducing the error associated with their estimations. As for the deadlocked dyads, the optimal selection of input components, if found, is not maintained for a sufficiently long period. Because L-agents are provided with lower levels of information and suffer the H-agent’s vacillations of choice, their performance does not provide information that is useful for H-agents. In fact, the H- and L-agents have become entrained in a vicious circle in which they confuse each other, and from which they cannot escape even after having accumulated a considerable amount of experience with the task (cf. the results of the simulation of the HL model over 100 epochs illustrated in Fig. S1).

Our main result, that the joint learning outcome is sensitive to initial learning experiences of the H-agents, which in turn depends on feedback from L-agents, is derived from a model where H-agents neither resort to sophisticated statistical reasoning, nor attempt to predict the behavior of L-agents. In the interest of defining boundary conditions of our results, we examined whether endowing H-agents with higher levels of sophistication might affect our conclusions. We examined two types of sophisticated behavior in which the H-agent: 1) is more patient, allowing L-agents to operate with lower learning rates and sample over longer spells, and more radically 2) transforms the joint learning problem into a pure statistical problem, i.e., forgoes learning about attractions, and instead samples each filter, estimate their expected performance, and use a statistical test (e.g., ANOVA) to, once and for all, choose the filter with highest performance. We examined both options and found that our main result holds even under increasing sophistication as described under 1), but is moderated under 2) (see Table S1 of SI for details). Given that the task environment is stable, 1) define rather sharp boundary conditions for our main result, while 2) defines a condition under which it is moderated.

## Discussion

In this paper, we combine laboratory experiments and computational modeling to advance our understanding of the decision processes underlying goal-oriented behavior in hierarchically organized interactions. Our multi-level HL learning model and experimental design capture the stylized features of interaction among human actors situated in hierarchical organizations, showing that such interaction structure can produce persistent differences in the overall learning performance. In addition, this is the first time in which joint hierarchical learning is investigated experimentally, and the mechanisms underlying its heterogeneity are unveiled.

We study experimentally how dyads of agents learn an interdependent task over time. Our design relies on the distinction between strategy and operational decisions that is common to most hierarchical interactions. Examples range from supervising students to managing the workflow of employees. This distinction implies that H- and L-agents operate at different time scales and can rely on different kinds of information. The distribution of the performance level (measured with the estimation error) achieved by dyads in the final stage of the experiment is not unimodal, suggesting that dyads can be clustered into two distinct groups–learning dyads and deadlocked dyads–that exhibit qualitatively opposite and significantly different patterns of learning. Whereas learning dyads continue to improve their performance and towards the end of the experiment approach optimal behavior, deadlocked dyads lose their adaptive capabilities and stop learning at a very early stage.

To better understand the dynamics that affect dyads’ performance, we develop a computational model of multi-level learning that reproduces the same architecture of interaction as in the experiment with human participants. We find that homogeneous artificial agents are sufficient to replicate, on multiple dimensions, the learning trajectories we observed experimentally. Based on the fitting of the aggregate trend, our model accurately predicts the dyads’ heterogeneity in the learning patterns. Simulation results show that the endogenous learning dynamics that emerge in the early stage of interaction are sufficient to account for the experimental results, and that these dynamics persist even in the long run.

Our main result is that the joint learning outcome is sensitive to initial learning experiences of the H-agents, which in turn depends on feedback from L-agents. We examined boundary conditions for this result, and found that neither increasing patience, nor applying a systematic order of sampling is sufficient to moderate it. Only if the joint learning problem is transformed to a statistical problem can the optimal filter be identified with a likelihood close to one, albeit at the cost of increasing sample size. Even if the required level of sophistication was present in practical instantiations of joint learning, it is unlikely that a sample size large enough to reliably identify the optimal filter would be feasible.

Overall, our results suggest that inferior performances of hierarchically interacting individuals may, at least in part, be caused by the emerging self-enforcing dynamics of joint learning that are inherent to hierarchical interactions. Given that learning processes are commonly situated in a hierarchical setting, erroneous explanations may be constructed for observed learning outcomes if the possible effects of hierarchical decision processes are not taken into account.

## Methods

### Experimental procedure

Forty-four participants (university students, 24 females, *M*_age_ = 24.9, *SD*_age_ = 2.50) were anonymously and randomly matched in 22 dyads, and faced a sequence of interdependent tasks (there were no data exclusions). Although the sample size was determined in advance based on the number of subjects typically used in learning studies in the psychology and economics literature (see, for example, the large set of classical learning studies considered in refs^20^ and^24^), the small sample size could be a limitation of the present study (see, however, the description in SI of a second similar study replicating our main results). Within each dyad, the two participants were randomly assigned the role of H- and L-agent (to keep the framing as neutral as possible, we labeled the two roles simply as “Player 1” and “Player 2”). The pairing and the roles were kept constant all throughout the experiment. Before the experiment started, participants were given a hard copy of instructions (reported in SI), which was read aloud by the experimenter. After this, participants were allowed to ask for any clarifications.

Participants were paid a show-up fee of 60 DKK (about $10), and had the opportunity to win an additional bonus of 60 DKK based on their accuracy in one epoch randomly selected at the end of the experiment. More in detail, the probability of winning the additional bonus was set equal to (100−%*E*(*t*)), where %*E*(*t*) is the average of percent estimation errors by the L-agent at epoch t, or to zero if %*E*(*t*) in the sampled epoch exceeded 100.

### Inputs’ target value and information filters

Each of the 100 inputs faced by the L-agent is a triplet of integers independently and randomly drawn from the uniform distribution over [0, 100]. The target value *y*^*i*^ of input *i* is the weighted linear combination of its three components. Formally, if input *i* is represented by the vector ${{\boldsymbol{x}}}^{i}=\{{x}_{1}^{i},{x}_{2}^{i},{x}_{3}^{i}\}$, then its target value *y*^*i*^ is:

1 $${y}^{i}=\sum _{j=1}^{3}{w}_{j}\cdot {x}_{j}^{i}$$

where ${\boldsymbol{w}}=\{{w}_{1},\,{w}_{2},\,{w}_{3}\}$ is the vector of weights. The vector ***w*** is not known to the dyads, and remains constant throughout the experiment. The L-agent estimates the target value of each input only knowing the value of two of its three components, because of the information filter applied by the H-agent. The H-agent can choose among three possible filters that hide one of the three input components (Fig. 2A).

### Timing of interaction and feedback

In our design, the H- and L- agents act sequentially, as schematized in Fig. 2A. The H-agent moves first and selects an information filter. After that, the L-agent faces five estimation tasks, in which she has to estimate, in sequence, the value of five different inputs (each input is a triplet of integers independently and randomly drawn from the uniform distribution over [0, 100], whose value is determined as in equation 1). After each estimation task, the L-agent receives as feedback the estimated and correct input value, and the corresponding percent error. After the fifth estimation task, the H-agent receives as feedback the average percent error by the L-agent in the previous five estimation tasks (cf. experiment instructions in the SI). Once feedback is sent to the H-agent, an epoch is concluded, and the process is repeated for 20 times (20 epochs), thus implying a total of 20 choices by the H-agent and 100 estimations by the L-agent. The decisions by the H- and L- agents were not temporally constrained.

### The HL learning model

The HL model reproduces the hierarchical architecture of the learning process occurring in the experiment with human participants. It consists of two interdependent modules–one for the L agent, and one for the H agent (Fig. 4). This modeling strategy facilitates pursuit of the main goal of the present paper, which is to understand how supervised (L-agent) and unsupervised (H-agent) learning processes combine in repeated hierarchical interactions.

Other modeling strategies, based on Bayesian criteria, have been suggested (e.g., ref.^32^) for the integration of information in dyads of agents. As our setting is substantially different (H- and L-agents see different information on different time scales), such approaches do not apply directly to our case. However, a simplified modeling approach inspired by Bayesian updating procedure is discussed and compared to our model in the SI.

#### The H-module

The model assumes that the H-agent associates each available choice alternative to a real value called *attraction* (or *propensity*). In our experiment, only three possible choices (the information filters ***f***_***i***_, i = 1,2,3) are available to the H-agent, corresponding to the three possible combinations of one hidden and two disclosed input components. The attractions *a*_*i*_, i = 1, 2, 3, determine the probability with which the H-agent will choose the information filter *i* via the following logistic rule:

2 $${p}_{i}=\frac{{e}^{\gamma \cdot {a}_{i}}}{{\sum }_{j=1}^{3}{e}^{\gamma \cdot {a}_{j}}},$$

where *γ* is a free parameter determining the sensitivity of the H-agent to differences among attractions. When *γ* = 0, the H-agent is insensitive to differences in attraction and picks any alternative with equal probability, whereas when *γ* tends to infinity, the H-agent behaves deterministically, preferring the alternative with the highest attraction.

The H-agent learns by updating its attractions. After epoch *t*, only the attraction corresponding to the selected filter *k*, *a*_*k*_, is updated according to the following:

3 $${a}_{k}(t+1)={a}_{k}(t)+\pi (t),$$

where $\pi (t)=max\{0,\,100\,-\, \% E(t)\}$, and where %*E*(*t*) is the average percent estimation error by the L-agent at epoch t.

#### The L module

We model the L-agent as being endowed with a set of receptor units, one for each input component, and one effector unit that provides as an output the estimation of the input value. The values of the input components are encoded in the receptor units. The effector’s output *o* is the weighted sum of the filtered component values. Formally, if the vector of weights is denoted with ${\boldsymbol{v}}=\{{v}_{1},\,{v}_{2},\,{v}_{3}\}$, the information filter applied by the H-agent with ${\boldsymbol{f}}=\{{f}_{1},{f}_{2},{f}_{3}\}$ (with two components equal to 1 and one equal to 0), and the vector of input components with ${\boldsymbol{x}}=\{{x}_{1},\,{x}_{2},\,{x}_{3}\}$, then the output ***o*** is defined as:

4 $$o={\sum }_{j=1}^{3}{v}_{j}\cdot {x}_{j}\cdot {f}_{j}.$$

Thus, the L module has the structure of a simple neural network usually referred to as *simple perceptron*^33,34^. Given the information filter ***f***, the L-agent learns by adjusting the weights *v* according to the following delta rule:

5 $${v}_{j}(t+1)={v}_{j}(t)+\lambda \cdot \delta (t)\cdot {f}_{j}\cdot {x}_{j},\,j=1,\,2,\,3.$$

where *δ*(*t*) is the error term, i.e., the difference between the input target value *y* and the evaluation ***o*** at trial *t*, that is $\delta (t)=y(t)-o(t)$. The initial weight values are independent random draws from the U[0, 1] distribution. The free parameter *λ* determines the speed of learning.

The delta rule has a rather intuitive description: Weights ***v*** are adjusted according to the error term ***δ***, but weights associated with larger input components get a proportionally higher adjustment. It can be shown that the delta rule leads to an adaptive process of gradient descent of the sum of squared error function^19^.

### Data Fitting

Our multi-level learning model has two free parameters, namely, the H-agent’s sensitivity to estimation error *γ*, and the L-agent’s learning rate *λ*. Since the attractions of H-agents were initially set to the same value (mimicking equal priors), this initial value is irrelevant (see eq. 2). We estimated the value of these parameters based on the two main aggregate measures of performance: The estimation error and the rate of disclosed information over epochs (i.e., the empirical data displayed in Fig. 3A,C).

We adopted the grid-search method for the estimation of parameters^20,24^. That is, for each combination of parameter values, we ran 20,000 artificial dyads and computed the average simulated curves for the estimation error and the rate of disclosed information over epochs. Then, for each statistic, we computed the mean square deviation (MSD) between the simulated trajectory and the empirical one, and the obtained MSD values were divided by the largest MSD value (ref.^35^ gives an axiomatic justification of the MSD-based parameter estimation). This normalization was necessary, as the two reference statistics are measured on different scales. The parameter estimates are the combination of parameter values that minimize the sum of the normalized MSDs. The best fit parameters that produced the data illustrated in Fig. 3 are 0.55 for the H-agent’s sensitivity *γ* and 1.3E-5 for the L-agent’s learning rate *λ*. The details of the explored parameter space are included in the SI.

### Statistical testing

All proposed statistical tests are two-tailed.

### Use of human participants

The reported experiments were conducted in accordance with the standards of the Faculty of Social Sciences at the University of Southern Denmark. Participation in the described experiments was conditional on the informed consent of each and every participant. The informed consent form, which outlines the participants’ rights as well as the conditions under which the experiment was run, was read, filled out, and signed by participants before the beginning of each experiment. Participants were adult volunteers, and the experiments described in this paper did not have any, potential or actual, negative impact on participants (such as stigmatization of particular social groups, political or financial retaliation, benefit-sharing, malevolent use, etc.). The reported experiments comply with the American Psychological Association’s Ethical Principles of Psychologists and Code of Conduct (available at http://www.apa.org/ethics/code/index.aspx); the Danish guidelines for research ethics in the Social Sciences (Vejledende Retningslinier for Forskningsetik i Samfundsvidenskaberne, available at http://ufm.dk/publikationer/2002/vejledende-retningslinier-for-forskningsetik-i-samfundsvidenskaberne); and the EU legislation on data protection and privacy (in particular, the Directive 95/46/EC on the protection of personal data). According to the Danish Act on Research Ethics Review of Health Research Projects, Part 4, Section 14, Number 2, this study was not subject to the evaluation of an ethical committee.

## Electronic supplementary material

Supplementary Information

## Data Availability

The data that support the findings of this study as well as the code developed for the simulations are available from the corresponding author upon reasonable request.
