# Supplementary material for: Hierarchical decision-making produces persistent differences in learning performance
Source: Sci Rep. 2018 Oct 25;8:15782. doi: 10.1038/s41598-018-34128-w (PMC6202344; doi:10.1038/s41598-018-34128-w)
Supplement: Supplementary file 1 — Supplementary Information [file 41598_2018_34128_MOESM1_ESM.docx]

**Hierarchical decision-making produces persistent differences in learning performance**

**Supporting Information**

Thorbjørn Knudsen^[[1]](#footnote-2)^, Davide Marchiori^a,1^, and Massimo Warglien^[[2]](#footnote-3)^

1. **More on Study 1 empirical and simulation results**
   1. **When does a dyad become deadlocked?**

According to our experimental data, learning and deadlocked human dyads do not display any significant differences in the first epochs, but after five epochs it is already possible to predict whether a dyad will fail or proceed on a successful learning trajectory.

The average estimation error and disclosed information rate measured over the first five epochs are not significantly correlated with the estimation error averaged over the last five epochs (Spearman’s rho = 0.21; S = 1394, p = .34 for the estimation error; Spearman’s rho = -0.15; S = 2040.80, p = .50 for the disclosed information rate). This is because the differences between the two groups in the early epochs of the experiment are very small. In the first five epochs, the estimation error is not significantly different across the two groups (25.17, SD = 5.10, for deadlocked dyads, and 22.21, SD = 5.71, for learning dyads; t[20] = 1.29, p = .21). Neither is there a difference across the two groups regarding the rate of disclosed information (0.68, SD = 0.07, for deadlocked dyads, and 0.69, SD = 0.07, for learning dyads; t[20] = 0.47, p = .64). In contrast, the average estimation error and disclosed information rate in the second block of five epochs are both significantly and highly correlated with the estimation error averaged over the last five epochs. For the estimation error, this correlation is 0.70 (Spearman’s rho; S = 526, p < .001), whereas for the disclosed information rate the correlation is -0.60 (Spearman’s rho; S = 2830.03, p < .01). In addition, the two measures are significantly different across the two groups (t[20] = 4.65, p < 0.001, for the estimation error; t[20] = 2.95, p = 0.008, for the disclosed information rate).

- 1. **Predicted asymptotic behavior**

The baseline simulation reported in the paper reproduces the results obtained for the duration of the experiment. We ran additional simulations to address the important question of whether the performance trajectories by learning and deadlocked dyads eventually converge to a common value. To this end, we simulated an experiment identical to the one described in the main text, but extended the duration of the simulation to include 100 epochs instead of 20. To allow for a meaningful comparison with the simulation results reported in the main text, learning and deadlocked dyads were determined by a median split based on the estimation error averaged over epochs 16-20. The results show that extended experience, gained over a longer duration of the learning process, does not affect the performance level of dyads once the learning process has converged (Fig. S1).

**Fig. S1.** Simulated Study 1 over 100 epochs. The performance trajectories for learning and deadlocked dyads are not converging to a common value even after 100 epochs. Thus, after the first few epochs in which dyads’ learning processes diverge, extended learning experience cannot override the self-reinforced dynamics that trap dyads into their performance group.

- 1. **Cognitive sophistication of the H-agent and the value of structured schemes of sampling**

We here present some simulations that further investigate the role of the H-agent’s unsupervised learning process. A natural question in this regard is whether enhancing the sophistication of the H-agent could improve dyadic performance. We can think of at least two different kinds of sophistication: One in which the H-agent is more “patient”, and one in which the H-agent implements a systematic sampling scheme that facilitates statistical decision making. Patient H-agents would act on longer time horizons (i.e., more than 20 epochs), be less sensitive to feedback, or allow a larger number of estimation tasks per epoch for the L-agents. Systematic H-agents would repeatedly sample each of the available options (filters), and then select the best option based on a statistical analysis of the observed data. It turns out that these sophistications neither eliminate performance heterogeneity, nor guarantee optimal performance. As described above, Fig. S1 reports the simulation of a patient H-agent who acts over 100 epochs compared to the 20 epochs used in our experiment with human participants. Fig. S2, panels A and B, reports the case in which the H-agent allow the L-agent a longer time horizon of 10 and 20 estimation tasks per epoch rather than the 5 tasks used in our experiment with human participants. Fig. S2, panels D and E, reports the case where the H-agent is initialized with a parameter of sensitivity to feedback that is either larger or smaller than the parameter estimated with from the experimental data.

A B

CDE

**Fig. S2.** Implementation of patient H-agents. Panels A and B report simulation data in which the H-agent allows the L-agent to conduct 10 and 20 estimation tasks per epoch (under the best-fit model parameters). Panels D and E show how increasing and decreasing the H-agent’s sensitivity parameter (and in this sense the speed of learning) affects performance. More in detail, Panel D shows that multiplying the sensitivity parameter, by a factor of 10, speeds up the convergence of the learning dyads (an effect that is similar to increasing the number of estimation tasks per epoch); Panel E shows how reducing the sensitivity parameter, by a factor of 10, maintains some level of variance even in the long-term performance.

Examining the effect of H-agents who apply systematic sampling schemes turned out to be particularly interesting. In this case, the H-agent transforms the learning problem into a statistical problem, which implies self-commitment to use each filter for a given number of epochs before applying an F-test to choose the filter with superior expected performance, i.e. the filter supplied by the L-agent, which comes with the lowest estimation error. Our simulations show that the effectiveness of this scheme is heavily affected by the sequence in which each filter is sampled: For each of the six possible sampling sequences, we computed the frequency with which an F-test is not significant, thus indicating no statistical differences in the goodness of the filters (see Table S1). Under the best-fit value for the learning parameter of the L-agent, even after having sampled each filter 32 times (thus well beyond the horizon of 20 epochs of our experimental setting), an F-test does not always recognize a difference across the average estimation errors associated to each filter (see the right-hand column of Table S1).

Why is it that a systematic statistical approach to identifying the optimal filter may fail? This is because the L-agent learns to apply weights from the application of a specific filter, and these weights will effectively define a set of priors that influence the L-agent’s learning path when the H-agent introduces a new filter. The order, or sequence, that filters are presented to L-agents therefore influences the L-agent’s learning process (this is why the problem we analyze cannot be equated to a k-armed bandit). This effect where the “shadow of the past” influences the L-agent’s learning path, and thereby the information that the H-agent submits to a statistical test both depends on the L-agent’s learning rate, and the number of samples the L-agent takes from each filter. This is because a high learning rate may lead to premature convergence on a suboptimal set of weights, which is the case when L-agents are first presented with filter 1 (worst filter), and then filter 3 (best filter), and finally filter 2 (see Table S1). Even when the sampling sequence does not decisively influence the outcome, the sequence with which L-agents are presented with the filters influences the number of epochs required to guarantee that the statistical test reliably identifies the optimal filter (see Table S1). Thus, even if H-agents do sample more systematically, the sampling order plays an important role as a source of heterogeneous adaptation.

**Table S1.** Systematic sampling by the H-agent.

| **Sampling sequence** | **best-fit** $\boldsymbol{\lambda}$ | | | | |
| --- | --- | --- | --- | --- | --- |
|  | **2 samples** | **4 samples** | **8 samples** | **16 samples** | **32 samples** |
| 1, 2, 3 | 66.95 | 26.65 | 7.42 | 0.38 | 0 |
| 1, 3, 2 | 69.36 | 24.58 | 5.7 | 3.78 | 9.74 |
| 2, 1, 3 | 69.81 | 38.14 | 25.17 | 12.2 | 1.77 |
| 2, 3, 1 | 76.38 | 36.29 | 19.79 | 11.37 | 2.12 |
| 3, 1, 2 | 81.35 | 55.34 | 17.83 | 0.13 | 0 |
| 3, 2, 1 | 83.59 | 58.37 | 24.1 | 0.23 | 0 |
|  | **best-fit** $\boldsymbol{\lambda}$ *** 2** | | | | |
|  | **2 samples** | **4 samples** | **8 samples** | **16 samples** | **32 samples** |
| 1, 2, 3 | 72.91 | 32.99 | 8.06 | 1.46 | 0.15 |
| 1, 3, 2 | 71.59 | 31.68 | 24.33 | 47.5 | 86.91 |
| 2, 1, 3 | 80.01 | 64.88 | 56.37 | 33.96 | 1.4 |
| 2, 3, 1 | 77.68 | 41.14 | 27.29 | 15.65 | 4.43 |
| 3, 1, 2 | 83.88 | 47.76 | 6.27 | 0 | 0 |
| 3, 2, 1 | 85.38 | 48.71 | 6.13 | 0 | 0 |
|  | **best-fit** $\boldsymbol{\lambda}$ **/ 2** | | | | |
|  | **2 samples** | **4 samples** | **8 samples** | **16 samples** | **32 samples** |
| 1, 2, 3 | 67.73 | 25.6 | 7.84 | 0.84 | 0 |
| 1, 3, 2 | 69.89 | 31.91 | 4.72 | 0.34 | 0.03 |
| 2, 1, 3 | 67.87 | 24.31 | 11.44 | 3.71 | 0.02 |
| 2, 3, 1 | 75.18 | 37.35 | 12.88 | 9.84 | 3.87 |
| 3, 1, 2 | 77.12 | 49.34 | 28.24 | 3.33 | 0 |
| 3, 2, 1 | 78.73 | 56.36 | 37.07 | 10.53 | 0 |

In these simulations, the H-agent transforms the learning problem into a statistical problem, which implies self-commitment to use each filter for a given number of epochs (2, 4, 8, 16, 32), collecting the data provided by the L-agent, and then applying an F-test test to choose the filter with superior expected performance. The parameter $\lambda$ tunes the speed of learning of the L-agent. For example, in the “4 samples” scheme and sampling sequence “1, 2, 3”, the H-agent samples four times Filter 1, four times Filter 2, and four times Filter 3. Filter 1 is the one that hides the input component with weight 0.9 (thus the “worst filter”), Filter 2 hides the input component with weight 0.5, and, finally, Filter 3 hides the input component with weight 0.1 (being thus the “best” filter). After having selected her available options according to the given sampling scheme, the H-agent runs an F-test to check for possible significant differences in the observed sequence of estimation errors by the L-agent to establish which filter is, on average, the best. The table reports the frequency (percentages over 10,000 simulations) of the cases in which the F-test gives a non-significant result at the 5% confidence level, indicating no statistical differences across the average estimation errors associated to the filters.

1. **Study 1: Further computational investigation**
   1. **Explored parameter space**

Table S2 reports the description of the parameter search space that was explored for the estimation of the HL model parameters in Study 1 and 2. Notice that the HL model has two free parameters, namely, the H-agent’s sensitivity to error (γ) and the L-agent’s learning rate (λ). The portions of the parameters’ space that have been investigated were suggested by previous computational studies that analyzed similar models (1-3).

**Table S2.** Explored parameter space.

| **Parameter** | **Initial value –> final value** | **Increment** |
| --- | --- | --- |
| H-agent’s sensitivity to error (γ) | 0.30 –> 2.20 | 0.05 |
| L-agent’s learning rate (λ) | 5.5E-6 –> 20.0E-6 | 0.5E-6 |

- 1. **Goodness of fit and predictive value of the HL model**

As described in the main text, parameters of the HL model were estimated on basis of the normalized sum of two indexes averaged over all 22 dyads: One index of performance (i.e., the estimation error), and one process index (i.e., the switch rate). These data are reported in panels A and C of Fig. 1, featured in the main text. The simulated data reported in the remaining panels of Fig. 1 are *out-of-sample* predictions, as these data were not used to estimate the model parameters, i.e. the predictions were based on variables that define a different sample space than the sample space defined by the variables used to estimate model parameters.^[[3]](#footnote-4)^

To assess the goodness of fit of the HL model, we studied the distribution of the obtained residuals. For both measures, the data show that the residuals computed for the fitted model are symmetrically and normally distributed, and their mean is not significantly different from zero (see Table S3 for the results of the tests). This implies that the HL model fits the experimental data without any systematic biases.

**Table S3.** Goodness of fit of the HL model: Analysis of the distribution of residuals.

|  | **Aggregate Indicators** | |
| --- | --- | --- |
|  | **Estimation error** | **Switch rate** |
| Symmetry of the distribution of residuals | Test statistic = 1.20  p = 0.21 | Test statistic = 0.979  p = 0.35 |
| Normality of the distribution of residuals | W = 0.968  p = 0.72 | W = 0.971  p = 0.80 |
| Difference from zero of the mean residual | t(19) = 0.850  p = 0.41 | t(18) = 1.738  p = 0.10 |

To test the symmetry of the distribution of residuals we used an *m-out-of-n bootstrap symmetry test* by Miao, Gel, and Gastwirth (2006), whereas to test the normality of the distribution of residuals we used a Shapiro-Wilk test.

To assess the predictive value of the HL model, we computed three different measures that are commonly used in the literature on time series forecasting. The first measure is the so-called Mean Forecast Error (MFE), which is defined as the mean of the residuals (see, for example, ref. 4). Values of the MFE close to zero do not indicate a perfect fit, but rather that the model does not produce a forecast bias (positive and negative errors cancel out). The mean squared deviation (MSD) is defined as the average of the squared residuals (see ref. 5). The MSD complements the MFE as it gives an overall idea about the magnitude of the error made by the model, but it does not provide any hint about the direction of the error. In the MSD, individual large errors are heavily penalized. The third measure we consider is Theil’s U-statistic (6,7), which is a (normalized) measure of forecast accuracy. Indicating with $y_{i}$ the observed $i$th data point and with $\hat{y}_{i}$ its estimate, the Theil’s U-statistic is defined as:

$$U=\frac{\left[ \sum_{i=1}^{n} \left( y_{i}-\hat{y}_{i} \right)^{2} \right]^{1/2}}{\left[ \sum_{i=1}^{n} {y_{i}}^{2} \right]^{1/2}+\left[ \sum_{i=1}^{n} {\hat{y}_{i}}^{2} \right]^{1/2}}$$

and is bounded between 0 and 1. The closer to zero is the U-statistic, the better the forecast, whereas the case of U = 1 (termed by Theil as “maximum inequality” between $y_{i}$ and $\hat{y}_{i}$) indicates either a negative proportionality, or that one of the variables is always equal to zero.

The computed values for these three described measures (reported in Table S4) are useful for comparing the accuracy of the fit with that of the out-of-sample predictions provided by the model; however, they are less informative when assessed in absolute terms.

**Table S4.** Quantifications of the fit and predictive value of the HL model.

|  | | **Fit/Prediction measures** | | |
| --- | --- | --- | --- | --- |
|  |  | **Mean Forecast Error (MFE)** | **Mean Squared Error (MSD)** | **Theil’s U-Statistic** |
| Fit | Aggregate  estimation error | 0.001  (0.30) | 0.00002  (2.38) | 0.0006  (0.18) |
|  | Aggregate  switch rate | 0.04 | 0.01 | 0.10 |
| Prediction | Estimation error:  Learning dyads | 0.00023  (0.07) | 0.00006  (5.67) | 0.0011  (0.33) |
|  | Estimation error:  Deadlocked dyads | 0.0017  (0.52) | 0.00006  (4.99) | 0.0008  (0.23) |
|  | Switch rate:  Learning dyads | 0.006 | 0.02 | 0.13 |
|  | Switch rate:  Deadlocked dyads | 0.09 | 0.02 | 0.11 |
|  | Aggregate rate of info disclosed | 0.04 | 0.003 | 0.03 |
|  | Rate of info disclosed:  Learning dyads | 0.003 | 0.002 | 0.03 |
|  | Rate of info disclosed:  Deadlocked dyads | 0.07 | 0.009 | 0.06 |

All three proposed measures of forecast performance are affected by changes in the scale of the data. Thus, to obtain homogeneous forecast measures, we normalized the estimation error by dividing it by the maximum possible estimation error (i.e., 300), obtaining a variable ranging in the interval [0, 1], as the switch rate and the rate of disclosed info. Between parentheses, we report the forecast measures for the non-normalized estimation error.

1. **Model comparison**

To clarify the partial contribution of the learning processes by the H- and L-agent, and test the robustness of the fitting and predictive power of the baseline model, we propose and compare six variants of the baseline HL model described in the main text. The first four variants enhance or decrease–one at a time–the degree of cognitive sophistication of the H- and L-agent assumed by the baseline model. In the fifth variant, the level of sophistication is enhanced for both the H- and L-agent. In the sixth variant, the H-agent’ learning rule implements a pseudo Bayesian approach for updating beliefs about the value of each filter. Table S5 summarizes the fitting and predictive performance of the baseline HL model and the six mentioned variants thereof.

**Table S5.** Results from the model comparison.

| **Model**  **(# of free parameters)** | **Best fit parameter values** | **Fit MSD**  **Est. Error**  **(20 datapts.)** | **Fit MSD**  **Switch rate**  **(20 datapts.)** | **Pred. MSD**  **Est. Error**  **(40 datapts.)** | **Pred. MSD**  **Switch rate**  **(40 datapts.)** |
| --- | --- | --- | --- | --- | --- |
| Baseline (2) | $\lambda=1.3E-5$  $\gamma=0.55$ | 0.0000265  (2.384) | 0.0148 | 0.0000592  (5.332) | 0.0225 |
| Variant 1:  Naïve L (1) | $\gamma=0.9$ | 0.00184  (165.326) | 0.0287 | 0.00207  (185.992) | 0.0380 |
| Variant 2:  Sophisticated L (2) | $\lambda(0)=1.15E-5$  $\gamma=0.55$ | 0.0000261  (2.349) | 0.0146 | 0.0000599  (5.389) | 0.0224 |
| Variant 3:  Naïve H (1) | $\lambda=1.4E-5$ | 0.0000333  (2.999) | 0.201 | 0.000127  (11.431) | 0.218 |
| Variant 4:  Sophisticated H (3) | $\lambda=1.25E-5$  $\gamma=0.45$  $\alpha=1.25$ | 0.0000254  (2.286) | 0.0157 | 0.0000593  (5.337) | 0.0236 |
| Variant 5:  Sophisticated H and L (3) | $\lambda(0)=1.35E-5$  $\gamma=0.35$  $\alpha=1.45$ | 0.0000243  (2.188) | 0.0134 | 0.0000580  (5.225) | 0.0202 |
| Variant 6:  Pseudo-Bayesian H (2) | $\lambda=1.15E-5$ | 0.0000332  (2.990) | 0.136 | 0.0000793  (7.141) | 0.153 |

The nested nature of the considered models precludes the use of an information criterion that penalizes the number of free parameters for a model comparison (14). Therefore, for each model we propose the mean squared deviation (MSD) obtained from the empirically observed data. MSD “fit measures” were computed on the 20 data-points of the aggregated trajectories. The MSD “prediction measures” were computed on the two trajectories (20 data-points each) for the learning and deadlocked dyads. Since the MSD measure is sensitive to the scale of the data, to obtain homogeneous forecast measures, we normalized the estimation error by dividing it by the maximum possible estimation error (i.e., 300), obtaining a variable ranging in the interval [0, 1], as the switch rate. Between parentheses, we report the forecast measures for the non-normalized estimation error.

*Variant 1: Naïve L-agent.* In this variant, the H- and L-agent are modeled as in the baseline model, except that the learning parameter $\lambda$ of the L-agent is set to 0. Under this constraint, the L-agent does not learn from experience, and its weights are never updated (see eq. 5 in the main text). Although this one-parameter model still reproduces persistent performance heterogeneity, the fit and prediction of the error curves are poorer than for the baseline HL model (see Fig. S3 and Table S5).

**A B**

**C D**

**Fig. S3.** Variant 1: Naïve L-agent. Fitted (panels A and C) and predicted (panels B and D) data.

*Variant 2: Sophisticated L-agent*. In this variant, the H-agent is modeled as in the baseline model, whereas for the L-agent the learning parameter $\lambda$ that appears in eq. 5 (main text) is replaced by a self-adjusting function. Dynamic learning rates allow for faster adaptation to surprising feedback, as well as for stabilization of behavior once the task has been learned.

**A B**

**C D**

**Fig. S4.** Variant 2: Sophisticated L-agent. Fitted (panels A and C) and predicted (panels B and D) data.

In this variant, $\lambda$ is function of the slope of the unsigned prediction errors (see related ideas in refs. 8 and 9). At trial *t*, the parameter $\lambda$ is computed as:

$\lambda\left( t \right)= \lambda\left( t-1 \right)+slope(t)\cdot\lambda\left( t-1 \right)$, [1]

where

$$slope\left( t \right)= \frac{\delta abs\left( t \right)-\delta abs\left( t-1 \right)}{\left( \delta abs\left( t \right)+\delta abs\left( t-1 \right) \right)/2}. [2]$$

The slope of the unsigned prediction errors (indicated with $\delta abs$, see also eq. 5 in the main text) is approximated by the difference between the current prediction error (i.e., $\delta abs\left( t \right)$) and the one observed in the previous trial (i.e., $\delta abs\left( t-1 \right)$), and normalized by their average to make it independent from the scale of payoffs. Positive slopes increase the value of $\lambda$, thus increasing the rate of learning and facilitating faster adaptation, whereas negative slopes decrease the value of $\lambda$, thus stabilizing the behavioral responses. This variant has two free parameters: the initial value $\lambda(0)$ of the learning parameter of the L-agent, and the H-agent’s sensitivity parameter $\gamma$. With respect to the baseline model, this variant improves the fit of the estimation error curve, but not its prediction; it also marginally improves both the fit and the prediction of the switch rate (see Fig. S4 and Table S5).

*Variant 3: Naïve H-agent.* In this variant, the H-agent only selects the filter associated with the largest propensity, obtained by setting the sensitivity parameter $\gamma$ in eq. 2 of the main text to $+\infty$ (computationally, we implemented this by setting $\gamma=1000$). The L-agent is instead as in the baseline model. The only free parameter of this model is the learning rate $\lambda$ that tunes the speed of learning of the L-agent (eq. 5 of the main text). Fig. S5 shows that whereas this model can replicate the heterogeneity of dyadic performance (but providing the second-worst fit of the estimation error curve), it fails to capture the behavior of the H-agent (see also Table S5). This is because reinforcements are always positive, which implies that the H-agent always selects the option that was randomly chosen (and that has been first reinforced) at the first epoch. In this sense, this variant inhibits the H-agent’s learning.

**A B**

**C D**

**Fig. S5.** Variant 3: Naïve H agent. Fitted (panels A and C) and predicted (panels B and D) data.

*Variant 4: Sophisticated H-agent.* In this variant, the L-agent’s model is as in the baseline, whereas the reinforcement rule for the H-agent (eq. 3 of the main text) includes one additional free parameter. Given that the H-agent has selected the *j*-th filter at epoch t, eq. 3 of the main text becomes:

$$a_{k}\left( t+1 \right)=a_{k}\left( t \right)+\alpha\cdot\pi\left( t \right), if k=j [3]$$

where $a_{k}$ is the attraction corresponding to filter $k$ ($k=1,2,3$), $\pi\left( t \right)$ is $max\left\{ 0, 100-\%E\left( t \right) \right\}$, $\%E\left( t \right)$ is the average percent estimation error by the L-agent at epoch *t*, and $\alpha$ is a free parameter defined over the positive real line. The parameter $\alpha$ tunes the learning rate of the H-agent, thus generalizing the baseline model (when $\alpha=1$, the two models are identical). It turns out that whereas this 3-parameter model ($\gamma$, $\lambda$, and $\alpha$) does marginally better in the fit of the aggregate estimation error curve, it does not outperform the baseline model in all the other computed measures of fit and prediction (Fig. S6 and Table S5). The best fit value of $\alpha$ implies a relatively faster learning pace with respect to the baseline model.

**A B**

**C D**

**Fig. S6.** Variant 4: Sophisticated H-agent. Fitted (panels A and C) and predicted (panels B and D) data.

*Variant 5: Sophisticated H- and L-agent.*  This variant puts together a sophisticated L-agent and a sophisticated H-agent as modeled in variants 2 and 4. Thus, variants 1-4 introduced earlier as well as the baseline HL model can be understood as simplifications of this fifth variant with sophisticated agents. This model is the best performing model, both in terms of fit and predictive power (see Fig. S7 and Table S5). However, such improvements in comparison to the performance of the baseline model should be assessed vis-à-vis the introduction of an additional free parameter. It is important to note here that the nested nature of the considered models precludes the use of an information criterion that penalizes the number of free parameters for a model comparison (10).

**A B**

**C D**

**Fig. S7.** Variant 5: Sophisticated H- and L-agent. Fitted (panels A and C) and predicted (panels B and D) data.

*Variant 6: Pseudo-Bayesian H-agent.* In this variant, the H-agent sequentially updates its beliefs about the average payoff from each filter, and best replies to the formed beliefs. The L-agent is as in the baseline. Specifically, given that the *j*-th filter has been selected at epoch *t* and in a total of $t_{j}$ epochs before *t*, the posterior mean is defined as:

$$\mu_{t_{j}+1}=\frac{t_{j}}{t_{j}+1}\cdot\mu_{t_{j}}+\frac{1}{t_{j}+1}\cdot\pi\left( t \right), [4]$$

where $\mu_{t_{j}}$ is the mean payoff over the previous $t_{j}$ selections of filter *j* (j$=1,2,3$), $\pi\left( t \right)$ is the observed payoff at epoch *t* (defined in terms of the accuracy $max\left\{ 0, 100-\%E\left( t \right) \right\},$ as in the previous variants). Eq. 4 describes an H-agent that makes sequential gaussian Bayesian updates of his estimation of the average performance of each filter (11,12). This model only approximates a process sequential Bayesian updating, trading off computational and conceptual simplicity with generality about assumptions (most importantly, stationarity holds only asymptotically), hence its “pseudo” label. This variant has two free parameters, namely the learning rate $\lambda$ of the L-agent and the value of initial priors $\mu_{j}(0)$ (all beliefs are initialized with the same prior). They are estimated with a grid search procedure. Simulation results show that, similarly to what observed for Variant 3, the best reply dynamics tends to inhibit too soon H-agent’s search. Therefore, although the model captures the main trend in the estimation error, it fails to capture the H-agent’s behavior (see Fig. S8 and Table S5).

**A B**

**C D**

**Fig. S8.** Variant 6: Pseudo-Bayesian H-agent. Fitted (panels A and C) and predicted (panels B and D) data.

1. **Study 2: The importance of setting the right decision premises**
   1. **Motivation**

In a second study, we analyze the importance of setting useful decision premises for the L-agent, which in our setting translates into providing the most informative input components (filter) for the L-agent. In Study 1 (described in the main text), the H-agent could only disclose two out of three input components for the L-agent. Whereas the L-agent’s limited processing and attentional capabilities justify this design, it may appear unnatural to assume that H-agents cannot provide L-agents with full information, i.e., the value of all input components. Interestingly, disclosing full information generates a peculiar tradeoff. On the one hand, the disclosure of full information allows the L-agent to learn the weights of all input components, and thereby deliver estimates (to the H-agent) that are unaffected by a systematic, uncontrollable error component. On the other hand, processing more information is costly, and it is conceivable that increasing the information load could make it harder for the L-agent to learn the appropriate response. For example, adding low-validity features may act as a distractor to the L-agent’s learning process (13). In general, a learning task that requires estimation of a larger number of parameters both reduces the statistical power of samples and the speed of learning–a classical problem in learning theory (14,15). Thus, the H-agent must assess the potential gains from providing more information against the cost of information processing, and the risk of confusing the L-agent.

In Study 2, we relax the constraint on the maximum number of input components that can be disclosed to the L-agent. Study 2 allows full disclosure of three components, whereas in Study 1, H-agents disclosed two out of three components. Thus, in the design of Study 2 the H-agent can present the L-agent with all three input components. However, such choice is costly: If in any given epoch the H-agent selects this option, an additional cost of 15 percent points is added to the cost of the average percent estimation error by the L-agent in that epoch. The H-agents were made aware of this additional cost at the beginning of the experiment. In all other respects, the experimental design is identical to that of Study 1. That is, the pattern of interaction, the feedback information provided to participants, the weights of input components, and the way input components were determined and input target values were computed were the same as in Study 1.

The experimental design of Study 2 may arguably be viewed as a better way of characterizing allocation of processing and attentional efforts in organizations. In contrast to individual human actors, organizations have more freedom to widen their attention span, although this choice inevitably implies some costs (16). For example, widening a firm’s attention to possibly add new processes/products usually comes at significant organizational costs, or as costs related to a decrease in the quality of the output.

Using the cost-scale defined above, the choice of providing full information to the L-agent is not optimal. Indeed, if the H-agent is already disclosing the two most informative input components (i.e., those with weights 0.9 and 0.5), then the average information gain obtained from disclosing all three input components is about 6.7%, a value that must be compared to the cost of an increase of the average percent error of 15 percent points. Thus, the marginal cost of introducing one more information source is (slightly) higher than its marginal benefit–provided that the L-agent has learned to estimate inputs correctly.

Even if disclosing all input components is not optimal in the long run, the H-agent has no clue about the benefits of the added information before experience is accumulated and learning unfolds. For example, an H-agent could strategically decide to disclose all three input components only in the first few epochs, with the purpose of providing the L-agent opportunity to learn and/or to gain information about the weight of the component that will be hidden subsequently. Therefore, it is not trivial to predict how the cost/benefit considerations by the H-agent are going to influence the decision to disclose more information, and, in turn, the performance of the L-agent. Indeed, as mentioned earlier, it might also be the case that disclosing the value of all three input components could have the adverse effect of confounding the L-agent instead of speeding up learning. Study 2 was run also to clarify this point.

A final, but no less important purpose of Study 2 is to test the robustness of the behavioral patterns observed in Study 1, particularly regarding the heterogeneity of performance and its dynamic causes.

- 1. **Participants and Payment**

Sixty-four students from the University of Southern Denmark (33 females, M_age_ = 25.2, SD_age_ = 3.33) that did not participate in Study 1 served as participants in this experiment. The sample size of 64 subjects and 32 dyads was determined in advance, and there were no data exclusions. Within each dyad, two participants were randomly assigned the role of H- and L-agent (to keep the framing as neutral as possible, we labeled the two roles simply as “Player 1” and “Player 2”). The pairing and the roles were kept constant throughout the experiment. Before the experiment started, participants were given a hardcopy of instructions (reported below), which was read aloud by the experimenter. After this, participants could ask for clarifications.

Participants were paid a show-up fee of 60 DKK (about $10), and had the opportunity to win an additional bonus of 60 DKK based on estimation accuracy in an epoch that was randomly selected at the end of the experiment. More in detail, the probability of winning the additional bonus was set equal to $(100 - \%E\left( t \right))$, where $\%E\left( t \right)$ is the average of percent estimation errors by the L-agent at epoch t, or to zero if $\%E\left( t \right)$ in the sampled epoch exceeded 100. Note that the average percent error $\%E\left( t \right)$ included the additional 15 percent points if, in the sampled epoch, the H-agent had chosen to disclose the full information.

- 1. **Study 2 experimental results**

Our analysis of the results focuses on the statistics that were considered in the analysis of data from Study 1. The measure of dyads’ learning performance (estimation error) shows a pattern very similar to that of Study 1, displaying a characteristic learning curve (Fig. S9A). The observed performance heterogeneity is also similar to what we observed in Study 1. As in Study 1, we used the median of the average estimation error over the last five epochs to split the 32 dyads into two groups of 16 dyads. The median value in Study 2 was 16.14, which is very close to the value of 16.07 observed in Study 1. Also in Study 2, the two performance groups (learning and deadlocked dyads) exhibit significantly different patterns of behavior: A two-way ANOVA with repeated measures factor shows that the group effect for the estimation error is significant (F[1, 30] = 18.5, p < .001), and so is the epoch effect (F[19, 570] = 9.04, p < .001), and the group*epoch interaction effect (F[19, 570] = 2.80, p < .001). These results show that also in Study 2, it is the group of learning dyads that drives the aggregate trend while deadlocked dyads only learn during the first few epochs (Fig. S9B).

A B

C D

E F

**Fig. S9.** Empirical and simulated results from Study 2. Panels A, C, and E show the main empirical and simulated measures of learning performance averaged over all 32 dyads. The aggregate empirical data displayed in A and C were used to fit the HL model’s parameters. Remarkably, under the same parameter values, the HL model is able to predict the learning heterogeneity observed empirically for the estimation error and the switch rate (B and D), as well as the rate of disclosed information that was not used to fit the model parameters (E and F). Error bars indicate the 95% normal confidence intervals.

The H-agent’s performance follows the familiar pattern from Study 1 (Fig. S9E and F). As for the switch rate (Fig. S9C and D), the group and the epoch effects in an ANOVA with repeated measure factor are significant (respectively, F[1, 30] = 11.78, p = .002, and F[18, 540] = 6.85, p < 0.001), whereas the group*epoch interaction is significant at the 10 percent confidence level (F[18, 540] = 1.60, p = .055). The trajectory for the rate of disclosed information shows a significantly increasing trend (ANOVA with repeated measures factor, F[19, 570] = 2.24, p = .002). As regards the rate of disclosed information, the learning and deadlocked dyads exhibit significantly different patterns of behavior (ANOVA with repeated measures factor, F[1, 30] = 9.64, p = .004).

H-agents select the opportunity to disclose all input components in 22.5% of the epochs. At the same time, full disclosure occurs more often in early epochs, and less frequently in later epochs (Fig. S10A and B). A two-way ANOVA with repeated measures factor shows that the effect of the epoch factor is significant (F[19, 570] = 1.96, p = .009). This suggests that H-agents might use full disclosure to facilitate L-agents’ initial learning, but subsequently they reduce the number of disclosed components to increase efficiency. However, this conjecture is not supported by the data as it appears that the L-agent’s performance is damaged by the disclosure of all three input components. To investigate this possibility, we compared, for each dyad, the percent estimation error that followed the disclosure of two and three features. In this analysis, we considered the percent estimation error rate (the actual feedback given to the H-agent) that excludes the 15 percent points cost of disclosing the third attribute. Over the 32 dyads, the percent estimation error after disclosure of the three features (54.5%, SD = 23.97) is higher than that under the partial disclosure of two features (32.82%, SD = 12.90), and this difference is significant (t[25] = 3.50, p = .002). This pattern is also maintained within the two subgroups of learning and deadlocked dyads. The percent estimation error after disclosure of three features (52%, SD = 21.16, for learning dyads; 57%, SD = 27.13, for deadlocked dyads) is higher than that that followed the disclosure of two features (28.9%, SD = 13.16, for learning; 36.8%, SD = 11.73, for deadlocked dyads). The difference is significant for both groups (t[12] = 2.70, p = 0.02, for learning dyads; t[12] = 2.20, p = 0.048, for deadlocked dyads).

A B

**Fig. S10.** Rate of disclosure of all three input components over epochs in Study 2. Panel A shows the data for the 32 dyads, whereas Panel B the data for the learning and deadlocked dyads separately. Error bars indicate the 95% normal confidence intervals.

Thus, full disclosure appeared to confuse L-agents rather than facilitate their learning process. This is also, indirectly, evident when comparing the (aggregate) estimation error over the last five epochs for Study 1 and 2: In Study 1 it is 14.38 (SD = 9.40), smaller than the value of 15.54 (SD = 7.64) observed in Study 2. Although this difference is not significant (t[52] = 0.50, p = 0.62), the difference between estimation error over the last five epochs for learning dyads in Study 1 (6.09, SD = 3.63) and in Study 2 (9.28, SD = 4.53) is significant at the 10% confidence level (t[25] = 1.94, p = 0.06).

- 1. **Study 2 simulation methodology and results**

The methodology we adopted in the computational analysis of Study 2 was the same as in Study 1. Also in this case, the simulation setup closely mirrored the experimental settings. The HL model used to describe Study 1 data was extended so that H-agents could provide L-agents with all three input components. To closely replicate the incentive structure of the experiment with human participants, the reinforcement of this option was set equal to $max\left\{ 0, 100 - \%E\left( t \right)-15 \right\}$ (where $\%E\left( t \right)$ is the average percent estimation error by the L-agent at epoch *t*).

The HL model’s parameters were estimated with the same procedure used in Study 1. That is, for each combination of parameter values, we computed the mean square deviation (MSD) between the empirical and simulated trajectories of the aggregated estimation error and rate of disclosed information. The MSDs were normalized, and the parameter estimates were those that minimized the sum of the normalized MSD for the two statistics. Also in this case, simulation results were averaged over 20,000 artificial dyads.

In this study, the model’s fit and prediction of the other performance measures (Fig. S9, panels C-F) appear to be worse than in Study 1. In many cases, the irregular shape of the empirical trajectories appears to reduce the statistical fit. This in turn might be the result of the higher variance in behavior induced by the “confusion” effect of full disclosure. However, in some cases the model underestimates the higher information disclosure in deadlocked dyads and, which generates more systematic departures from the empirical data. For example, the empirical rate of disclosed information averaged over the last five epochs is 0.91 (SD = 0.07) for learning dyads, and 0.77 (SD = 0.13) for deadlocked dyads, whereas the simulated rate is 0.91 (SD = 0.08) for learning dyads, and 0.65 (SD = 0.13) for deadlocked dyads. The model also fails to predict the dramatic drop of switch rates by H-agents in learning dyads. Thus, it seems that the model fails to capture some component of H-agents’ behavior that make them slightly “smarter” than plain reinforcement learning.

1. **Experimental Instructions for Study 1**

At the beginning of this experiment, you will be paired with another participant. The pairing will be random and anonymous. Once you will have been paired, each of you will be randomly assigned to the role of **Player 1** or **Player 2** (your partner will be assigned the role complementary to yours). All throughout the experiment, you will play together with the same partner, and in the same role.

In this experiment, you and your partner will jointly have to accomplish two tasks (explained below in detail): 1) handling a sequence of estimation tasks; 2) managing information so you can deliver estimates as accurately as possible. According to your assigned role, you will address one of these two tasks, and your partner the other. Specifically, Player 1 will have to manage the relevant information that will be useful for Player 2 to make accurate estimates.

This experiment includes many **epochs**, and each epoch includes many **trials**. At the first trial of each epoch, Player 1 will have to decide what information to disclose to Player 2. Then, given this information, Player 2 will face a sequence of estimation tasks. After this, a new epoch will start, and so on until the end of the experiment.

**The *estimation* tasks.** In each estimation task, Player 2 will be given some information about three factors, that is, three (integer) numbers drawn randomly and independently from 0 to 100. You will have to estimate the **combined value** of these three factors (see figure below, right-hand side). Player 2’s task will not be trivial for the following reasons:

1. The combined value of the three factors is given by their **weighted sum**. This means that if, for example, the factors are 35, 50, and 82, then their combined value will be equal to (w_1_ * 35 + w_2_ * 50 + w_3_ * 82). Therefore, in order to compute the exact combined value, you should know the values of the three **weights w_1_, w_2_, and w_3_**.
2. Player 1 and 2 will not be informed about the exact values of the weights. Both players will only know that a) weights are the same across epochs, and b) they are numbers between 0 and 1 (but they *do not* necessarily sum up to 1).
3. Player 2 will only be informed about the values of two out of the three factors.

Please, keep also in mind these important facts:

1. The combined values can range between 0 and 300;
2. Each weight is associated to the same factor all throughout the experiment;
3. According to their weights, factors can **contribute differently** to their combined value.

**The *information managing* task.** As mentioned, Player 2 will only be informed about the values of two factors out of three (see figure below, right-hand side). At the beginning of each epoch, Player 1 must decide which factors will be disclosed to Player 2 in that epoch: Only the values of the two disclosed factors will be shown to Player 2, whereas the hidden one will be marked with “NA” (that is, “Not Available”). Starting from the second epoch, Player 1 will receive feedback about the **average** **percent error rate^[[4]](#footnote-5)^** by Player 2 in the previous epoch (see figure below, left-hand side).

Player 1’s task will not be trivial as he/she will have to decide what information can be more useful for Player 2. Player 1 can make his/her decisions based on Player 2’s average percent error rate in the previous epoch, thus without knowing the values of the weights.


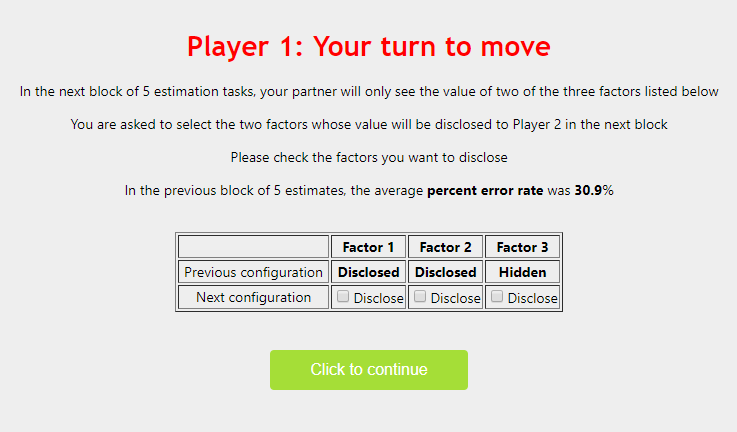

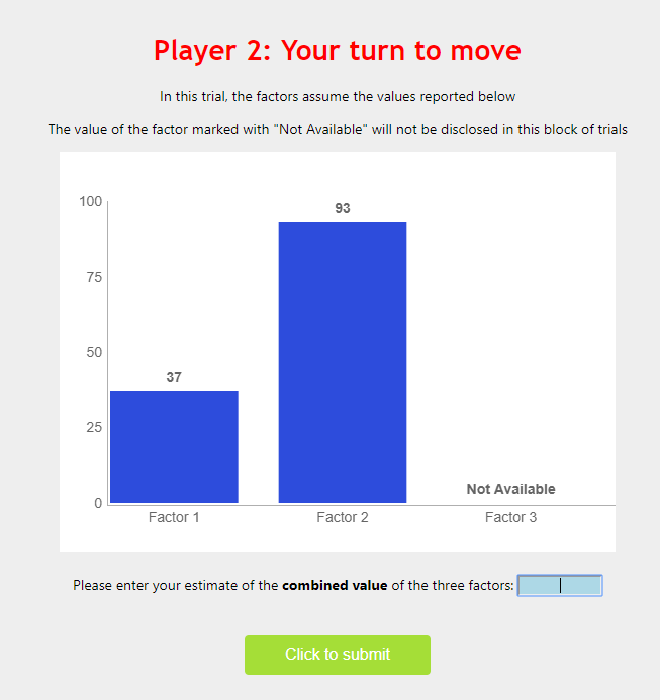


Both your own, and your partner’s final payoff will depend upon the accuracy of the estimates made by the one of you who acted as Player 2, given the information made available by Player 1.

At the end of the experiment, an epoch will be randomly selected and you and your partner will win a 60 Kroner voucher each with probability equal to the average accuracy rate (that is, 100 - avg. percent error rate) in the sampled epoch. Moreover, both of you will receive an additional voucher of 60 Kroner for your participation.

During the experiment, you will not be allowed to communicate (nor verbally or in any other way) with the other participants.

**References**

1. Erev, I., & Roth, A.E. Predicting how people play games: Reinforcement learning in experimental games with unique, mixed strategy equilibria. *Am. Econ. Rev.* **88**, 848-881 (1998).

2. Marchiori, D., & Warglien, M. Predicting human interactive learning by regret-driven neural networks. *Science* **319**, 1111-1113 (2008).

3. Marchiori, D., & Warglien, M. Neural network models of learning and categorization in multigame experiments. *Front. Neurosci.* **5**, 1-14 (2011).

4. Butler, K.C., & Lang, L.H. The forecast accuracy of individual analysts: Evidence of systematic optimism and pessimism. *J. Account. Res.* **29**, 150-156 (1991).

5. Selten, R. Axiomatic characterization of the quadratic scoring rule. *Exp. Econ*. **1**, 43-61 (1998).

6. Theil, H. *Economic Forecasts and Policy* (*North Holland, 1965*).

7. Bliemel, F. Theil’s forecast accuracy coefficient: A clarification. *J. Mark. Res.* 10, 444-446 (1973).

8. Krugel, L.K., Biele, G., Mohr, P.N.C., Li, S-C., & Heekeren, H.R.. Genetic variation in dopaminergic neuromodulation influences the ability to rapidly and flexibly adapt decisions. *Proc. Natl. Acad. Sci. USA* **106**, 17951-17956 (2009).

9. Stieglitz, N., Knudsen, T., & Becker, M. Adaptation and inertia in dynamic environments. *Strat. Mgmt. J.* **37**, 1854–1864 (2016).

10. Burnham, K.P., & Anderson, D.R. *Model Selection and Multimodal Inference* (*Springer-Verlag, 2002*).

11. Box, G.E.P., & Tiao, G.C. *Bayesian Inference in Statistical Analysis* (*John Wiley, 1992*).

12. Lee, P. M. *Bayesian statistics: an introduction* (*John Wiley, 2012*).

13. Kruschke, J.K., & Johansen, M.K. A model of probabilistic category learning. *J. Exp. Psychol. Learn.* **25**, 1083–1119 (1999).

14. Vapnik, N.V. *Statistical learning theory* (*John Wiley and Sons, 1998*).

15. Cucker, F., & Smale, S. On the mathematical foundations of learning. *B. Am. Math. Soc.* **39**, 1–49 (2001).

16. Gifford, S. Allocation of entrepreneurial attention. *J. Econ. Behav. Organ.* **19**, 265–284 (1992).

1. Strategic Organization Design unit and Danish Institute for Advanced Study, University of Southern Denmark [↑](#footnote-ref-2)
2. Department of Management, Ca’ Foscari University of Venice

   ^1^ To whom correspondence should be addressed. Email: davmar@sam.sdu.dk. [↑](#footnote-ref-3)
3. We do not apply a split-sample methodology, but rather derive sample spaces on the basis of different variables obtained from a given sample. [↑](#footnote-ref-4)
4. Defined as: $\frac{\left| estimate-true value \right|}{true value}*100$ [↑](#footnote-ref-5)
